# Vincristine-Induced Peripheral Neuropathy (VIPN) in Pediatric Tumors: Mechanisms, Risk Factors, Strategies of Prevention and Treatment

**DOI:** 10.3390/ijms22084112

**Published:** 2021-04-16

**Authors:** Silvia Triarico, Alberto Romano, Giorgio Attinà, Michele Antonio Capozza, Palma Maurizi, Stefano Mastrangelo, Antonio Ruggiero

**Affiliations:** Pediatric Oncology Unit, Fondazione Policlinico Universitario A. Gemelli IRCCS, Università Cattolica Sacro Cuore, 00168 Rome, Italy; silviatriarico@libero.it (S.T.); albertoromano90.ar@gmail.com (A.R.); giorgio.attina@policlinicogemelli.it (G.A.); micheleantoniocapozza@gmail.com (M.A.C.); palma.maurizi@unicatt.it (P.M.); stefano.mastrangelo@unicatt.it (S.M.)

**Keywords:** vincristine, chemotherapy-induced peripheral neuropathy, pediatric cancer, molecular mechanisms, risk factors, prevention, treatment

## Abstract

Vincristine-induced peripheral neurotoxicity (VIPN) is a very common side effect of vincristine chemotherapy among pediatric patients with cancer. Neuropathy may be sensory, motor and/or autonomic, with consequent reduction, delay or discontinuation of vincristine-chemotherapy, but also pain, disability, reduced quality of life of patients and an increase in medical costs. Vincristine acts out its antineoplastic function by altering the normal assembly and disassembly of microtubules, with their consequent mitosis block and death. Vincristine leads to VIPN through a complex mechanism of damage, which occurs not only on the microtubules, but also on the endothelium and the mitochondria of nerve cells. Furthermore, both patient-related risk factors (age, race, ethnicity and genetic polymorphisms) and treatment-related risk factors (dose, time of infusion and drug–drug interactions) are involved in the pathogenesis of VIPN. There is a lack of consensus about the prophylaxis and treatment of VIPN among pediatric oncologic patients, despite several molecules (such as gabapentin, pyridoxine and pyridostigmine, glutamic acid and glutamine) having been already investigated in clinical trials. This review describes the molecular mechanisms of VIPN and analyzes the risk factors and the principal drugs adopted for the prophylaxis and treatment of VIPN in pediatric patients with cancer.

## 1. Introduction

Vinka alkaloid (VAs) drugs (vincristine, vinblastine, vinorelbine, vindesine and vinflunine) are a class of microtubule-targeting agents that interfere with the continuous mitotic divisions and cell growth of cancer cells [1]. Vincristine is one of the most used VAs in pediatric patients with cancer and it has been incorporated in several poly-chemotherapy regimens for acute lymphoblastic leukemia (ALL), lymphomas, neuroblastoma, sarcomas and central nervous system tumors. However, neurotoxicity is a severe and dose-limiting side effect of vincristine and it may produce delay or discontinuation of the treatment. Vincristine may cause peripheral, progressive (almost distally to proximally) and symmetric nerve damage, due to microtubule structure disruption, inflammatory processes and axonal dysfunction [2].

Clinical patterns of VIPN may be divided into three categories:

− sensory neuropathy: paresthesia, numbness, impaired touch sensitivity/temperature recognition/vibration, neuropathic pain, jaw pain;
− motor neuropathy: extremity weakness, walking difficulties, deteriorated reflexes and fine motor abilities, impaired balance, muscle cramps;
− autonomic neuropathy: constipation, paralytic ileus, incontinence, urinary retention, orthostatic hypotension [3,4].

Despite the lowly penetration of vincristine through the blood–brain barrier into the central nervous system [5], central nervous system and cranial nerves toxicities have been described, such as ptosis, ophthalmoplegia, color vision deficiency, blindness and blurred vision, diplopia, strabismus, ocular muscle paresis, limited mobility of jaw and facial muscles, hearing loss and ototoxicity, reduced tongue movements, stridor and persistent cough [6], syndrome of inappropriate secretion of antidiuretic hormone (SIADH) [7] and encephalopathy with seizures/disorientation/aphasia/hemiplegia [8]. In their retrospective study about the incidence of vincristine neurotoxicity among 103 children with acute lymphoblastic leukemia (ALL), Nazir et al. described the development of VIPN in 19 patients, but also a relatively common incidence of autonomic neuropathy (one bradycardia, one tachycardia, five abdominal pain and constipation) and cranial nerves toxicities (one unilateral hearing loss, two severe life-threatening cranial nerve involvement with bilateral ptosis and recurrent laryngeal nerve involvement). Signs and symptoms can appear within a week of the start of therapy and can remain unchanged for up to 12 months following dose reduction, or persist for years beyond treatment conclusion [6].

Several tools have been used for assessing and measuring VIPN in pediatric oncology patients. The Common Terminology Criteria for Adverse Events (CTCAE) can be used also for the assessment of peripheral neuropathy, but it shows a poor sensitivity in detecting motor and sensory neuropathy [9]. The Total Neuropathy Score-Pediatric Vincristine (TNS-PV), developed by Lavoie-Smith et al., seems to be more precise for the assessment of VIPN in pediatric oncology patients older than 6 years. The TNS-PV consists of an interview-based questionnaire and a standardized physical examination (testing of vibration and temperature sensibility, muscle strength and deep tendon reflexes) [10]. Moreover, physical examinations performed by specifically trained physicians and nerve conduction studies using somatosensory evoked potentials can also be adopted for the assessment of VIPN, although they may be invasive and painful. Recently, the pediatric-modified Total Neuropathy Score (ped-mTNS) was developed, which consists of a quick, inexpensive, non-invasive, interview-based questionnaire and physical examination, with greater psychometric characteristics compared to the other tools for the assessment of pediatric VIPN. The ped-mTNS has been evaluated in North-American and Dutch children with cancer aged 5–18 years [11].

VIPN may result in dose reduction, delay or discontinuation of vincristine-chemotherapy, but also in pain and disability, reducing the quality of life of patients and increasing medical costs [12,13].

In this review, we analyze the pharmacokinetics and pharmacodynamics of vincristine, with a focus on the mechanisms, risk factors and strategies of prevention and treatment of VIPN in pediatric patients treated for cancer. We have searched for papers dedicated to VIPN in the pediatric age, performing a Pubmed-based retrieval of articles using the search terms “vincristine”, “Vinka alkaloid”, “neurotoxicity” and “peripheral neurotoxicity”, matched with “children”, “childhood” and “pediatric”. After the original search, we used filters to select articles available in the English language and articles with available full texts. The final search retrieved 85 articles, of which 43 were exclusively related to pediatric patients.

## 2. Pharmacokinetics and Pharmacodynamics of Vincristine

Vincristine and the other VAs are compounds with a complex molecular structure, consisting of an indole nucleus and a dihydroindole nucleus linked by a CeC bridge. They are administered intravenously and are subsequently distributed bound partly to plasma proteins and partly to platelets [14]. The penetration of vincristine into the cells occurs through various mechanisms, including passive diffusion and active transport systems dependent on energy and temperature [15]. Once into the cells, it carries out its antineoplastic function by binding to the microtubules and inhibiting their functions [1].

Microtubules are intracellular proteins, which constitute the cytoskeleton of eukaryotic cells together with microfilaments and intermediate filaments. They consist of two fundamental subunits called α-tubulin and β-tubulin, associated with each other forming polarized cylindrical structures in which α-tubulin constitutes the negative terminal (−) and β-tubulin the positive terminal (+). A third member of the tubulin family, γ-tubulin, plays a role in microtubule nucleation and assembly [16]. The microtubules polymerize and depolymerize continuously inside the cell, by adding tubulin to the + end and subtracting it from the—end, producing a continuous centripetal renewal phenomenon called the treadmilling process. This process of continuous assembly takes place through the hydrolysis of GTP, which is bound to the β-tubulin and is essential for numerous vital cell functions, including cell division. During mitosis the microtubules assemble, forming the mitotic spindle, which allows the replication and segregation of the chromosomes in the two daughter cells [17].

**Figure 1:**
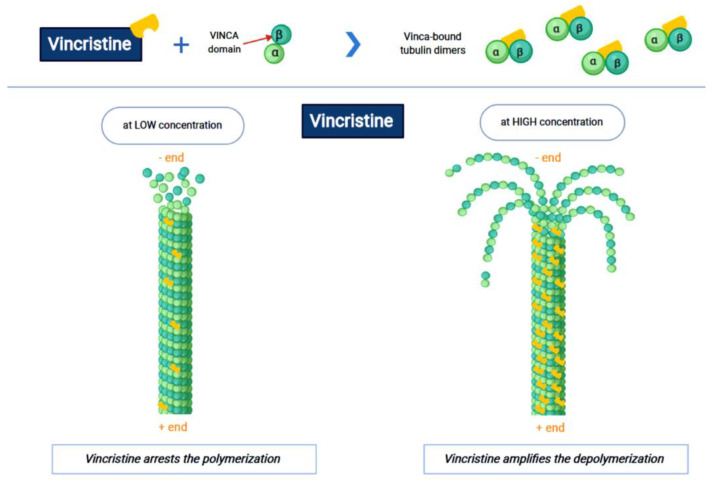
The first part of the figure shows the interaction between vincristine and the tubulin dimers. The second part shows the vincristine concentration-dependent action: the arrest of polymerization at low dose and amplification of depolymerization at a high dose.

Vincristine acts as an inhibitor of the treadmilling process, by the link to the tubulins, which prevents the formation of the microtubules and consequently of the mitotic spindle. In this way, cell division is blocked and the cell dies [18]. In particular, vincristine and the other VAs interact with tubulin dimers by binding to approximately sixteen–seventeen different specific binding sites, known as the vinca domain [19]. Once the binding site is reached, VAs have a concentration-dependent action: at low concentrations, VAs prevent microtubules from elongating, by binding to the + end and preventing GTP from binding to β-tubulin [20]; at high concentrations, they promote the depolarization of microtubules, as explained in Figure 1.

Once the formation of the mitotic spindle is blocked, the cell goes into apoptosis with or without p53 activation [21]. The VAs damage does not only concern the mitosis, but also all the other processes that involve the microtubules, such as the inhibition of axon transport, secretion processes, structure disorders and impairment of platelet functions [22]. Since the action of the drug is related to its ability to act on tubulins, its concentration in tissues changes according to the tubulin isotype expressed in the cells [23]. The P-glycoprotein-mediated multidrug resistance to vinca alkaloids, whose function is regulated by calmodulin and intracellular calcium concentration, operates the active efflux of the drug from the cells [15].

Vincristine has poor oral bioavailability and is administered intravenously as vincristine sulfate, which is a vesicant. After intravenous administration, vincristine rapidly distributes extensively into most body tissues, with poor penetration across the blood–brain barrier (BBB) and into the central nervous system (CNS). However, it is very neurotoxic and fatal if administered intrathecally, because it produces quickly serious leptomeningitis and ventriculitis [5]. Vincristine metabolism is performed in the liver by the cytochrome p450 CYP3A enzyme system, particularly by CYP3A4 and CYP3A5. Vincristine has a half-life of 85 h and it is eliminated primarily via the biliary route and excreted in the feces; consequently, great attention should be paid in the presence of hyperbilirubinemia [1]. The kidney eliminates a very small amount of the drug [24]. Vincristine has little myelosuppressive effects and is usually given even to leukopenic and thrombocytopenic patients.

## 3. Pathogenesis of VIPN

The pathogenesis of VIPN is strictly connected to the mechanisms through which vincristine carries out its antineoplastic function. As previously mentioned, vincristine acts primarily by altering the normal assembly and disassembly function of microtubules, with consequent mitosis block and cell death. In addition to this, all other activities that involve microtubules are inevitably compromised. Inside the neurons, microtubules are not involved in the constitution of the mitotic spindle, since these cells are not in active replication. Microtubules are abundant in neurons, because they make up the skeleton of axons and dendrites, giving them their specialized morphologies [1,25].

In addition to acting like bearing struts, microtubules are the main long-distance railways along which proteins and organelles are actively transported in both directions within the axons and dendrites. This allows for the normal transmission function of the nerve impulse. By binding to microtubules, vincristine causes changes of neuron shape and stability, preventing the retrograde and anterograde axonal transport with consequent Wallerian degeneration, which consists of the reabsorption of the distal segment of a nerve after it has been damaged, and also causes the alteration of nerve impulse transmission and neuronal death [26].

Microtubules play a fundamental role in the myelination of nervous fibers, because they are essential constituents of oligodendrocytes. As recently demonstrated by Lee et al., vincristine destabilizes the microtubules, altering the oligodendrocyte structure and function, with consequent abnormal myelination and loss of peripheral sensory fiber [27].

In addition to its direct structural and functional damage of nerve cells, vincristine also induces the expression of integrins (immune markers) on the surface of endothelial cells, allowing macrophages to express the CX3CR receptor for the adhesion to the endothelium and the migration into the nervous tissue. This process causes the production of reactive oxygen species (ROS), which act as a chemical mediator for immune-neuronal communication by activating transient receptor potential TRPA1 channels (functionally expressed by the axons of sensory neurons) and evoking pain [28].

Furthermore, vincristine facilitates the binding of the Signal Transducer and Activator of Transcription 3 (STAT3) to the CXCL12 gene promoter, determining the upregulation of C-X-C Motif Chemokine Ligand 12 (CXCL12) in dorsal horn ganglia. CXCL12 is a member of the integrin family and acts as a ligand of CXCR4 (CD184, C-X-C chemokine receptor type 4); CXCR4 increases the intracellular concentration of calcium, resulting in the approaching of T-lymphocytes and monocytes. This series of events leads to the onset of an inflammatory process in the peripheral nervous system, with consequent worsening of the neurological damage [29].

The third mechanism of peripheral neuropathy is the damage to the mitochondria [30]. It has been recognized that vincristine can influence the movement of Ca^2+^ across the mitochondrial membrane, reducing both the quantity and the rate of Ca^2+^ absorption and decreasing its efflux [31]. The modification of the mitochondrial absorption and concentration of Ca^2+^ alters the mitochondrial function [32], with consequently increased exocytosis of neurotransmitters [33] and release of ROS [26]. These changes lead to reduced neuronal excitability and glial function, activating apoptosis. In support of this damage hypothesis, Flatters et al. reported a great incidence of swollen and vacuolated mitochondria with disrupted cristae localized at the periphery of the organelle [34].

**Figure 2:**
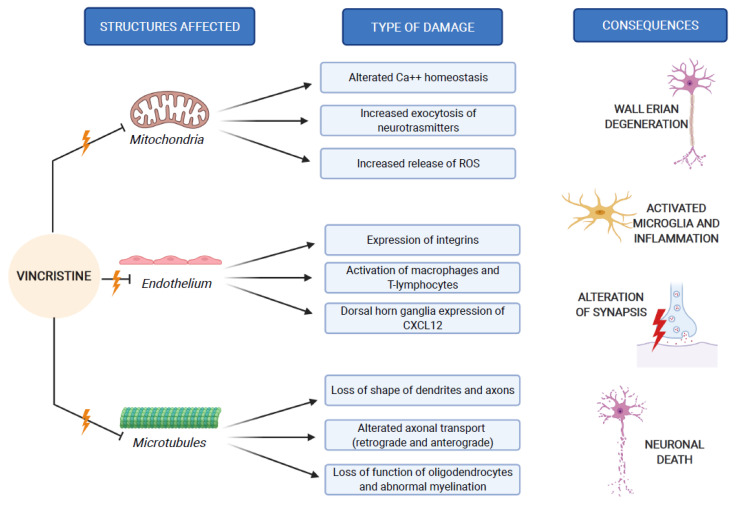
VIPN pathogenesis, which involves mitochondria, endothelium and microtubules of nerve cells.

These three mechanisms of neurological damage influence and amplify each other, strongly conditioned by patient-related risk factors and treatment-related risk factors, as we explain below. Figure 2 synthesizes the VIPN pathogenesis.

## 4. Patient-Related Risk Factors for VIPN

Age, sex and ethnicity represent the three patient-related factors that may influence the occurrence of VIPN. The available data do not completely clarify their influence on the appearance of VIPN. A recent review conducted by van de Velde et al. highlighted the discrepancy of the data available in the literature about the role of age on VIPN pathogenesis [35]. As they pointed out, younger children may be at greater risk of VIPN, due to incomplete maturation and myelination of the peripheral nervous system, and in fact children with the hereditary demyelinating form of Charcot–Marie–Tooth disease seem be more predisposed to VIPN [36]. As previously said, vincristine exerts its direct action on oligodendrocytes, by altering their function [28]. In young subjects this may cause abnormalities in the myelination process, leading to VIPN. On the other hand, age may influence pharmacokinetic variables and vincristine metabolism. Young children have a greater and quicker ability to metabolize vincristine than older ones; consequently, dose adaptation in younger children might prevent vincristine toxic plasma levels. Nevertheless, incomplete maturation and myelination of the peripheral nervous system and quicker vincristine metabolism in younger children are two factors that balance each other, with consequent divergent results regarding age as a risk factor for VIPN [37,38].

Of the 58 drafts evaluated by van de Velde et al., no clear evidence was identified regarding the influence of gender on the incidence of VIPN, while ethnicity seemed to play a significant role [35]. Ethnicity can influence the functionality of the cytochrome p450 3A (CYP3A) family, which plays a main role in the metabolism of vincristine. CYP3A4 and CYP3A5 are the two components of the CYP3A family primarily responsible for the metabolism of vincristine [39,40,41,42]. Reduced CYP3A5 activity is more frequently observed in the Caucasian population and it causes a reduced elimination rate of vincristine, leading to a greater risk of VIPN occurrence [43,44,45,46,47].

As evidenced by Madsen et al., other gene polymorphisms may influence the occurrence of VIPN [4]. Among these, CEP72 anomalies seem to facilitate the occurrence of VIPN, as described in several studies, although exhaustive data are not yet available [48,49]. CEP72 is a centrosomal protein essential for microtubule formation. Its lower expression increases the occurrence of microtubule inhibition, through a mechanism distinct from vincristine [40]. In the presence of CEP72 anomalies, vincristine binds to the VAs binding site on the microtubules and blocks the formation of microtubules through CEP72, amplifying the risk of VIPN [50].

Other gene polymorphisms that could facilitate the appearance of VIPN are those related to ABCC1 and ABCB1, SLC5A7 and TTPA genes. ABCC1 and ABCB1 encode for two ATP-binding cassettes, whose function is important in the excretion of vincristine from the cells and their polymorphism may improve vincristine damage. SLC5A7 encodes for a membrane transporter that carries choline within acetylcholine-secreting neurons. TTPA encodes for a protein responsible for the transport of alpha-tocopherol into the cells and its mutation causes a form of ataxia with isolated vitamin E deficiency, associated with peripheral neuropathy. Therefore, polymorphisms affecting SLC5A7 and TTPA are not directly involved in the damage caused by vincristine, but represent a predisposition to the onset of peripheral neuropathy [51].

Li et al. conducted a meta-analysis of genome analyses from two independent cohorts: Pediatric Oncology Group (POG) ALL trials and a multicenter study based at Indiana University in children with ALL. They identified two single-nucleotide polymorphisms (SNPS) significantly associated with VIPN: rs1045644 and rs7963521. Rs1045466, located on chromosome 14, is associated with the coagulation factor C homology (COCH) gene, whereas rs7963521, located on chromosome 12, is related to the regulation of chemerin plasma levels [52]. Moreover, in either of the two cohorts of their analysis, SNP in the CEP72 gene was not associated with severe VIPN, as opposed to what was stated by Stock et al. [48].

Micronutrient (vitamin B12, folate and iron) deficiencies and low BMI have been investigated as non-genetic causes of VIPN in children with ALL, but a correlation was not found [53]. Furthermore, in their study among 49 pediatric patients affected by B-ALL, Sajdyk et al. demonstrated that obesity is significantly associated with the development of VIPN. This may be explained by a major release of pro-inflammatory cytokines from the adipose tissue that may enhance vincristine’s neurotoxicity, but also by the possible storage of vincristine in the adipose tissue with consequent longer exposure to the peripheral nerves over time [54].

## 5. Treatment-Related Risk Factors for VIPN

Prolonged treatments and higher single doses of vincristine seem to be related to increased occurrence and severity of VIPN in adult patients, providing validation for a maximum vincristine dose of 2 mg [4,55]. The studies about the association between dose and VIPN in pediatric patients are lacking conclusive results and greater vincristine doses are not exactly associated with peripheral neuropathy in pediatric patients. However, the recommended dose is 0.05–0.065 mg/kg in infants and 1.5 mg/sqm in children, with a maximum of 2 mg/dose and a minimum of a week interval between each dose [35,56].

In their retrospective study among children with low-grade gliomas treated with carboplatin and vincristine, Rosca et al. demonstrated that VIPN is more developed during the induction phase, when the administrations of vincristine are more closely, suggesting an association between the increased risk of VIPN and the intensity of vincristine infusion [57]. Regarding the duration of vincristine administration, currently, bolus injections over 1–5 min are the standard for pediatric protocols. In a systematic review, van de Velde et al. showed that vincristine bolus injection increased the inter-compartmental clearance of the drug, which was significantly associated with VIPN. Thus, prolonging vincristine infusion from push injections to one-hour infusions may be a good strategy for reducing the risk of VIPN [35]. Recently, van de Velde et al. conducted a randomized controlled trial (the VINCA trial) among children with ALL, Hodgkin’s lymphoma, nephroblastoma, medulloblastoma, rhabdomyosarcoma and low-grade glioma treated with vincristine administered as a one-hour infusion or push injection. They did not find a different incidence of VIPN between the group of patients treated with a one-hour infusion of vincristine and those who received push injection. Furthermore, when concomitant azole antifungals were adopted, the incidence of VIPN was lower in the one-hour group than in the push group [58].

As said before, vincristine is metabolized by CYP3A4 in the liver. In pediatric patients, it is frequently described as the association between VIPN and concomitant interacting treatments with CYP3A4 inhibitors [59]. The azole antifungals are strong CYP3A4 inhibitors and neurotoxic themselves, even in the absence of other neurotoxic chemotherapy; consequently they should be avoided during vincristine regimens [60,61].

Among azoles, a relatively smaller neurotoxicity has been seen with fluconazole, which is a weaker CYP3A4 inhibitor than other azoles and should be preferred as antifungal prophylaxis when using vincristine. Fluconazole seems to be safer than other azoles when administered in association with vincristine, especially when used as antifungal prophylaxis. Fluconazole has a dose-dependent effect on the CYP450 enzyme system, so the risk of neurotoxicity is more consistent when used at the therapeutic dose [62].

Itraconazole is a stronger inhibitor of CYP3A4 than posaconazole and voriconazole, which are more potent inhibitors than fluconazole [63].

Lin et al. report the case of a child affected by acute lymphoblastic leukemia and treated with posaconazole for a mucormycosis infection. This patient developed severe toxicities with neuropathic pain and constipation from the association of posaconazole with vincristine chemotherapy [64]. The worst interaction was observed with itraconazole, a triazole that may cause remarkable and sometimes life-treating neurotoxicity, because of the inhibition not only of CYP3A4, but also of the P-glycoprotein efflux pump, with consequently enhanced vincristine intracellular concentrations [65,66,67]. Pana et al. collected 26 cases of children affected by ALL, who received azole treatment (with itraconazole, posaconazole or voriconazole) and developed neurotoxicity, with a high incidence of autonomic neuropathy (abdominal pain, constipation and paralytic ileus). A prompt withdrawal of the drug (especially of itraconazole) is mandatory in the case of azole-induced neurotoxicity, to avoid severe and life-threatening risks. The treatment with posazonazole, voriconazole and itraconazole should be interrupted almost 24 h prior to and after the administration of vincristine, if alternative antifungal therapies are contraindicated or not applicable. Dose-reduction of vincristine should be considered, but data from the literature demonstrate that withholding or reducing doses of vincristine may not result in a fast improvement of neurotoxicity [68].

Moreover, aprepitant and fosaprepitant are employed as antiemetic drugs, but due to their moderate inhibition on CYP3A4, they can produce important drug interactions [69]. Edwards et al., in their study, demonstrated a greater incidence of VIPN in patients with non-Hodgkin’s lymphoma who received aprepitant/fosaprepitant as an antiemetic regimen. Thus, during vincristine regimens, benefits and risks of their use as an antiemetic drug should be individually evaluated [70].

Table 1 summarizes the treatment-related risk factors for VIPN in children.

## 6. Strategies for Prevention and Treatment of VIPN

Currently, despite several drugs having been investigated also in pediatric oncologic patients, universally shared strategies for VIPN prevention and treatment are missing.

Gabapentin has been widely adopted for the management of pain associated with VIPN. Anghelescu et al. retrospectively reviewed the use of gabapentin in prophylaxis and treatment in children with ALL who developed neuropathic pain. Gabapentin was used after the first episode of pain associated with VIPN and as prophylaxis for subsequent episodes, at a starting dose of 5–10 mg/kg/day with a maximum of 50–70 mg/kg/day [71]. However, they did not find any significant evidence that gabapentin may reduce the recurrence of pain associated with VIPN better than opioids, highlighting the need for prospective randomized studies to elucidate the value of gabapentin regimens for prevention or treatment of neuropathic pain during treatment of childhood leukemia [71]. More recently, they also performed a randomized, double-blind, placebo-controlled trial on 51 pediatric patients who developed VIPN during the treatment for ALL, that were divided into two treatment arms: gabapentin plus opioid versus placebo plus opioid. Gabapentin plus opioid was not associated with a better analgesic efficacy than therapy with placebo plus opioid [72].

Pyridoxine (vitamin B6) seems to produce neuroprotection in a murine model with lethal VIPN and pyridostigmine (an acetylcholinesterase inhibitor) has been used for enhancement of intestinal motility in patients with reduced gastrointestinal motility [73,74]. Firstly, Müller et al. described that treatment with pyridoxine and pyridostigmine produced the full recovery of bilateral ptosis in a 2-year-old boy affected by synovial sarcoma and treated with vincristine [75]. Then, Akbayram et al. showed four cases of VIPN in children treated for acute lymphoblastic leukemia and the complete resolution of symptoms after 1–2 weeks of treatment with 150 mg/sqm/day of pyridoxine and 3 mg/kg/day of pyridostigmine, which was well-tolerated by patients without any side effects [76]. Recently, Köker et al. evaluated the features of VIPN and the effectiveness of pyridoxine plus pyridostigmine therapy in children with acute lymphoblastic leukemia; they studied 23 patients with acute lymphoblastic leukemia and VIPN and administered to 21 of them an oral dose of pyridoxine 150 mg/sqm/day and pyridostigmine 3 mg/kg/day for 3 months, observing an improvement in symptoms and the absence of side effects [77].

Glutamic acid is an excitatory neurotransmitter that seems to produce a protective activity from VIPN [78]. Bradfield et al. performed a randomized placebo-controlled, double-blind trial on the use of glutamic acid in 250 pediatric patients affected by Wilms’ tumor, rhabdomyosarcoma, non-Hodgkin’s lymphoma and ALL, who developed VIPN. Glutamic acid was administered before the first vincristine dose at a dose of 250 mg for body surface area < 1 sqm and 500 mg for body surface area ≥ 1 sqm. They found that glutamic acid was effective for the prevention of VIPN only in patients 13 years or older, but not in pre-adolescent patients [79]. In their randomized single-blinded placebo-controlled clinical trial, Mokhtar et al. evaluated the role of glutamic acid (administered at a dose of 1.5 g daily on the day before or on the day of the first dose of VCR) for preventing VIPN in pediatric patients with acute lymphoblastic leukemia, non-Hodgkin’s lymphoma patients and Wilms’ tumor. They discovered a reduction in neurotoxicity in the group treated with glutamic acid, which was well tolerated without adverse side effects [80].

In animal models, glutamine has been recognized to upregulate circulating nerve growth factor (NGF) mRNA, the levels of which are reduced in patients that undergo chemotherapy with neurotoxic agents [81]. Furthermore, pilot laboratory and human studies suggest that glutamine may improve microtubule formation and stability [82]. Sands et al. conducted a double-blind, randomized, placebo-controlled trial to investigate the efficacy of glutamine in the prevention of the progression and/or in the resolution of VIPN, in pediatric patients affected by non-Hodgkin’s lymphoma, Ewing sarcoma, Wilms’ tumor and rhabdomyosarcoma. Glutamine was administered at a dose of 6 g/sqm twice daily (up to a maximum of 10 g/dose) for 21 days. They found a statistically significant improvement in sensory function and overall quality of life in the group supplemented with glutamine [83]. Table 2 summarizes the principal drugs studied in clinical trials for the prophylaxis and treatment of VIPN in pediatric oncologic patients.

## 7. Conclusions

VIPN is a common side effect of vincristine treatment in pediatric oncologic patients and its pathogenesis seems to be multifactorial, related to patient-related risk factors (age, race, ethnicity and genetic polymorphisms) and treatment-related risk factors (dose, time of infusion and drug–drug interactions). The recognition of risk factors would allow clinicians the prompt identification of patients at higher risk, helping clinicians to manage them in the most appropriate and personalized way. Several molecules (such as gabapentin, pyridoxine and pyridostigmine, glutamic acid and glutamine) have been investigated for the prophylaxis and/or treatment of VIPN in pediatric oncologic patients. Furthermore, there is a lack of consensus about guidelines for the management of VIPN in this setting of patients, and future studies are required for evaluating novel preventive and therapeutic approaches.

## Figures and Tables

**Table 1:** Treatment-related risk factors for VIPN in children.

Risk Factor	Mechanism	Reference
Dose	Higher dose of vincristine could facilitate VIPN	[34,55]
Administration schedule	Dosing closely may increase the risk of VIPN	[36]
Method of administration	Prolonged administration could reduce the risk of VIPN compared to IV bolus administration	[34]
Concomitant use of other drugs	Azoles, aprepitant and fosaprepitant inhibit CYP3A4, increasing the risk of VIPN	[59,60,61,62,63,67,68,69]

**Table 2:** Principal drugs investigated for prophylaxis and treatment of VIPN in children.

Drug	Dosage	Patients Age (in Years)	Outcome	Reference
Gabapentin	5–10 mg/kg/day (max 50–70 mg/kg/day)	1–18	No evidence of superiority over opioids for reducing or preventing VIPN pain	[72]
Pyridoxine	150 mg/sqm/day	2–13	Complete resolution of symptoms of VIPN	[76]
Pyridostigmine	3 mg/kg/day	2–13	Complete resolution of symptoms of VIPN	[76]
Pyridoxine and Pyridostigmine	150 mg/sqm/day and 3 mg/kg/day	2–10	Significantly improvement of symptoms	[77]
Glutamic acid	250 mg daily for BSA * < 1 sqm, 500 mg daily for BSA ≥ 1 sqm		Prevention of VIPN only in patients aged 13 years or more	[79]
	1.5 g daily (on the day before or on the day of the first dose of VCR)	3–18	Reduced occurrence of VIPN	[80]
Glutamine	6 g/sqm twice daily for 21 days	5–21	Improvement in sensory function and QoL **	[83]

* BSA: body surface area. ** QoL: quality of life.
